# Supplementary material for: Tinnitus and occupational noise exposure among informal generator technicians in Nigeria: A pilot cohort study
Source: PLOS Glob Public Health. 2026 Feb 26;6(2):e0005032. doi: 10.1371/journal.pgph.0005032 (PMC12944749; doi:10.1371/journal.pgph.0005032)
Supplement: S1 Table — (DOCX) [file pgph.0005032.s002.docx]

**List of raw data legend for Tinnitus study**

| ParticipantID | Serialized identification of participants |
| --- | --- |
| Gender1Male2Female | Gender code (1-male; 2-female) |
| Age | Age of participant in years |
| PlaceofworkLGA/ward | Place of work LGA/ward |
| Marital Status | Marital Status of participants |
| EducationLevel | Highest level of education attained |
| Ethnicity1Hausa2Igbo3Yoruba4othersspecify | Ethnicity of participants (Hausa, Igbo, Yoruba, others-specify |
| Hoursworkdayhrs | Daily work hours of participants (hrs) |
| HouseholdIncomemonth | Monthly income of participants (grouped) |
| HearingLeftEar | Self-reported hearing loss - Left Ear |
| HearingRightEar | Self-reported hearing loss - Right Ear |
| HearingAidUse0No1Yes | Hearing Aid use by participant (0-No; 1-Yes) |
| Self-reported tinnitus | Presence of tinnitus in participant |
| DurationofExposure (Years) | Number of years working with generators |
| LeftEarExamination0NoIssue1Abnormal | Otoscopic examination of left ear (0-No issue; 1-Abnormal findings: wax, FB, exudate/discharge) |
| RightEarExamination0NoIssue1Abnormal | Otoscopic examination of Right ear (0-No issue; 1-Abnormal findings: wax, FB, exudate/discharge) |
| Avg hearing Threshold at normal freqs (R Ear-dB) | Puretone threshold value for Right Ear at speech frequencies (dB) |
| Avg hearing Threshold at normal freqs (L Ear-dB) | Puretone threshold value for Left Ear at speech frequencies (dB) |
| OAE_rawData_Right | Otoacoustic emission result for right ear (Pass/Fail) |
| OAE_rawData_Left | Otoacoustic emission result for left ear (Pass/Fail) |
| OAE_Status (pass/fail) | Binary coding for OAE Test outcome (0-Fail; 1-Pass) |
| AdviceGiventoParticipante.g.ReferProtectMonitor | Advice given to participant following assessments e.g.Refer/hearing protection required/Monitoring |
| Avg_Hearing_thresold_Combined | Mean hearing threshold per participant |
| Familyhistoryofdeafnesshearingloss1yes2No | Family history of deafness (1-Yes; 2-No) |
| Monthly_raw_Income (₦) | Raw Monthly income of participants |
| Income_Per_Month_coded | Income coded as per range of earning |
| RE_HL_coded | Right hearing loss (Code= 1-normal hearing; 2-abnormal thresholds) |
| LE_HL_coded | Left hearing loss (Code= 1-normal hearing; 2-abnormal thresholds) |
| Grade_HL | Grade of hearing loss. Normal (0-25dB) hearing; 1- Mild (26—40) HL; 2- Moderate (41-55) HL; 3-Moderately severe (56–70) HL; 4-Severe (71-90) HL; 5-Profound (>90) HL |
| Age_Groups_D_Code | Age groups coded: 0= (14-25), 1= (26-35), 2= (36-45), 3= (46-55), 4= (≥56) |
| Age_Cat | Age Category with 3 age levels: 0= Young (14-25), 1= Middle (26-45), 2= older (≥46) |
| PTAv_both_ears_Prevalence | Coding for Puretone averages to assess prevalence. 1- Normal hearing (0-25dB); 2- Hearing loss (≥26dB) |
| Tinnitus_present2_coded | Binary coding for presence of Tinnitus (0-No; 1-Yes) |
| Marital_Status2 | Coding for marital status: 0-Not married, 1-Married) |
| Av_Monthly_income2 | Income coded 0= <₦10,000, 1= ≥₦10,000 |
| DailyExposure_Hours2 | Daily noise exposure. Coded as 0- <8 hrs; 1- ≥8hrs |
| EducStatus_2 | Educational status- 0= (No formal education); 1= Primary; 2= secondary & above |
| Years_in_Occupation2 | Coding for exposure years. |
| Audiometry_Impairment/Status2 | Final binary Coding for Audiometric status of participants. 0-Normal hearing (0-25dB); 1- Hearing loss (≥25.50dB) |
| Family_Deafness2 | Coding for family history of deafness. 0- no history; 1- positive family history. |
