# Supplementary material for: Tinnitus and occupational noise exposure among informal generator technicians in Nigeria: A pilot cohort study
Source: PLOS Glob Public Health. 2026 Feb 26;6(2):e0005032. doi: 10.1371/journal.pgph.0005032 (PMC12944749; doi:10.1371/journal.pgph.0005032)
Supplement: Attachment — Submitted filename: Point-by-point response_Reviewer_Editor_comments_Tinnitus.pdf [file pgph.0005032.s006.pdf]

Manuscript Title: Tinnitus and Occupational Noise Exposure among Informal Generator Technicians: Insights from a Nigerian Pilot Study [PGPH-D-25-02022]

Dear Joanna Tindall,

We thank you and the reviewer for your thoughtful and constructive feedback on our manuscript. We have carefully considered all comments and revised the manuscript accordingly. We have provided below a point-by-point response to the major concerns raised:

1. Male-only participants

We acknowledge that our sample consisted exclusively of male participants. This reflects the occupational demographics of informal generator technicians in Northern Nigeria, where the profession is overwhelmingly male-dominated. We have clarified this in the Methods section as well.

2. Lack of confounder control

We recognize that confounding factors such as recreational noise exposure, mental health status, and medication use were not captured in our original questionnaire. We have acknowledged this limitation in the Discussion section and recommended future studies to include these variables.

3. Methodological limitations

We have expanded the Discussion section to elaborate on limitations including the cross-sectional design, small sample size, use of self-reported tinnitus without standardized tools, and potential sensitivity issues with the portable OAE device.

4. Language clarity

We have revised the manuscript for improved clarity, grammar, and readability throughout.

5. Data availability

We have updated the Data Availability statement to indicate that the dataset is made publicly available as supplementary information to this manuscript, in compliance with PLOS data sharing policies.

We appreciate the opportunity to revise our manuscript and believe the changes have strengthened its clarity and scientific contribution. We look forward to your further consideration.

Sincerely,

Abdulazeez Ahmed (Corresponding Author)

On behalf of all the co-authors
