# Supplementary material for: Tinnitus and occupational noise exposure among informal generator technicians in Nigeria: A pilot cohort study
Source: PLOS Glob Public Health. 2026 Feb 26;6(2):e0005032. doi: 10.1371/journal.pgph.0005032 (PMC12944749; doi:10.1371/journal.pgph.0005032)
Supplement: Attachment — Submitted filename: Response to Reviewers_Tinnitus_PGPH-D_02022 R1 (2).pdf [file pgph.0005032.s007.pdf]

Manuscript Title: Tinnitus and Occupational Noise Exposure among Informal Generator Technicians: Insights from a Nigerian Pilot Study [PGPH-D-25-02022 R1 (2)]

Dear Emma Campbell,

We thank you and the reviewers for your thoughtful and constructive feedback on our manuscript. We have carefully considered all comments and revised the manuscript accordingly. We have provided below a point-by-point response to the major concerns raised:

Reviewer #1:

1. Emphasize the pilot nature of the study in the abstract and conclusion to avoid overgeneralization.
   Response: This has now been adjusted accordingly, the title now has pilot cohort study added.
2. Clarify limitations: self-report tinnitus measure, absence of direct noise level recordings, male-only sample.
   Response: Statement clarifying all these has now been included in the limitation (Discussion section).
3. Strengthen the methods section by briefly justifying why no standardized tinnitus questionnaire was used.
   Response: Standardized tinnitus questionnaire/instruments were not used because a validated/local (Hausa-language) version was not available, and adaptation was beyond the scope of this study. We have also clarified this in the methods section.
4. Refine the discussion of psychosocial factors (marital status, stress, etc.)—frame these as hypotheses for future study rather than strong conclusions.
   Response: We have agreed to expunge this from the discussion section since the variables involved i.e. marital status, etc., was associated with tinnitus in the crude analysis only, and this effect was attenuated after adjusting for age and other covariates, therefore was not considered a robust predictor.

Reviewer #1: MAJOR COMMENTS

1. The manuscript lacks a comprehensive baseline table of participant demographics.
   Response: We have now included a detailed demographics table (Table 1) summarizing age, marital status, education, income, etc. Providing a clearer epidemiological profile of the cohort.
2. The current tables are repetitive with the Results text and often do not provide additional information or are so short as to be better reported simply in the text (i.e., Table 2, 3). Table 4 appears to be partially redundant with Figure 1 and does not report the proportions of each group reporting tinnitus.
   Response: We have restructured the Results section to flow from demographics → age prevalence → PTA/OAE × tinnitus cross-tabulation → regression analysis. Redundant tables were merged, and figures (LOESS curve, forest plot) were added to enhance clarity.
3. Three-dimensional bars graphs are not appropriate for publication so Figure 1 should be re-made as a flat stacked bar graph, with Ns and proportions clearly labeled. Forest plots illustrating the results of the regressions would also be helpful.

Response: We have expunged this 3D bar chart. A forest plot for regression has now been included as figure 2.

4. Ensure that all factual statements or claims about what is said in the literature are backed up by appropriate citations (e.g., lines 146-148, lines 238-240, etc.)
   Response: Appropriate citations have now been added to the specified sections.
5. Methodological details are scant and need to be expanded as listed below. An example informed consent form and examples of all assessment instruments should be included in the Supplementary Materials given non-standard items were used.
   Response: Informed consent form and assessment instruments are now included in the Supplementary Materials. Methodological details have now been improved upon.
6. Was any counseling or education provided to participants? Were they made aware of the study results? Provided with hearing protection?
   Response: Yes, there were provided, we have now added a dedicated statement in the Methods (Community/Participant Engagement) subsection to clarify these procedures.
7. The lack of an association with age is very surprising (given that it is a strongly established risk factor for tinnitus, especially in the context of noise exposure) and may be due to a lack of statistical rigor. How do the authors explain this finding? There should be a table tabulating tinnitus prevalence by age groups (e.g., decades) and plot it (age on x, % with tinnitus on y). Then fit a locally smoothed curve (e.g., LOESS) of prevalence vs. age to see shape. What did a logistic regression of age say (Model: $\text{logit}[P(\text{tinnitus}=1)] = \beta 0 + \beta 1 \cdot \text{Age}$). You could report odds ratio per 10-year increase (scale age/10 so β1 is per decade).
   Response: We appreciate the reviewer's detailed feedback regarding the role of age. We have now conducted and incorporated additional descriptive and graphical analyses to better characterize the relationship between age and tinnitus in this population. As recommended, we re-scaled age in decades and report the odds ratio for each 10-year increase. Although the adjusted association remained nonsignificant, the direction of effect was positive and consistent with established literature. An explanation has been added to the Results & Discussion sections.

Reviewer #2: SPECIFIC COMMENTS

Abstract:

1. The demographics of the cohort need to be better described (for example, age is missing).

Response: We have revised the Abstract to include the age range (14–57 years) and mean age of participants, thereby providing a clearer demographic profile.

Introduction

2. (Line 58–59): This wide range needs context from the smallest to largest proportion. Please specify what type of tinnitus you are referring to.

Response: We have clarified that the study assessed *subjective, non-pulsatile tinnitus*. The prevalence range cited has been contextualized by specifying the lowest and highest reported proportions from previous studies.

3. Line 73: What is the typical dB of the generators? Was this measured at any point (or cite the literature)?

Response: We have added ambient noise measurements (95–100 dB(A)) recorded during the study, and cited relevant literature confirming generator noise levels could exceed 85 dB(A).

4. Line 82: Why is this called a pilot study instead of a cohort study?
   Response: We have clarified in the Introduction and Discussion that this is a *pilot cohort study*, reflecting both its exploratory nature and modest sample size.

Methods

5. Please define the type of tinnitus you are assessing (subjective non-pulsatile?) and who administered all testing/instruments along with their expertise. Was testing on-site or at an institution?
   Response: We specified that *subjective, non-pulsatile tinnitus* was assessed using a structured questionnaire. Audiometric and OAE testing were conducted in a sound-proof booth by trained audiologists with >5 years' experience at our health facility.
6. Line 109: There is a statement that the cohort is representative of the field population but how is not stated. Please expand.
   Response: We expanded the Methods to explain that participants were randomly selected from union registries across four districts, proportionally to size, ensuring representativeness of the field population.
7. Line 111-112: The informed consent forms are not in the Supplemental Materials and should be included. How was consent obtained from minor (parents)? Depending on the journal requirements, the IRB approval may need to be mentioned in the Methods.
   Response: Informed consent forms have now been included in the Supplemental Materials. For minors (<18 years), parental consent and participant assent were obtained. Ethical approval from the Kano State Ethics Committee and Bayero University Health Research Ethics Committee has been explicitly stated in the Methods and also previously attached as supplemental material.
8. Line 112-113: It is unclear why people over age 57 years were excluded as presbycusis starts early in noise-exposed populations. A better approach is probably to not exclude by age, but include age as a variable in more detailed analyses to understand its impact.
   Response: We revised the Methods to clarify that exclusion was intended to minimize confounding by presbycusis. However, age was also analyzed as a continuous variable in regression models.
9. Line 121: What was the size of the registries randomly sampled from?
   Response: We have added that the union registry contained approximately 250 members, from which 73 participants were ultimately enrolled.
10. Line 125: Table 1 also includes mention of collection of family history of deafness. Those methods and definitions need to be fully described in the Methods section. Was it limited to immediate family? Genetic or acquired deafness? Self-report or diagnosis by physician?
    Response: We clarified that family history referred to immediate relatives, self-reported, and included both genetic and acquired deafness.
11. Line 128: What instrument was used to assess tinnitus history? All instruments should be included in the Supplemental Materials.
    Response: A structured interviewer-administered questionnaire was used; the instrument has now been included in the Supplemental Materials.
12. Line 131: Who administered the PTA and OAE tests? It seems you have the granularity to report more detailed information for the PTA tests (e.g., by frequencies) and I suggest you do so. It would be important to understand any differences in high vs. low frequency hearing loss. Additionally, no results of the otoscopic exams are reported.

Response: PTA and OAE were administered by certified Audiometricians with >5 year's experience. PTA thresholds and otoscopic exam findings are part of another study/manuscript, I am afraid we are unable to include these here.

13. Line 138-139: For OAE testing, please define what "fail" versus "pass indicates (what are the parameters?)

    Response: We defined OAE pass as a signal-to-noise ratio ≥6 dB at ≥3 frequencies; fail was below this threshold. A "pass" is considered only when both ears passed. Any unilateral or bilateral failure was considered indicative of cochlear dysfunction (OAE Fail) captured in the methods section.

14. Line 141: How were ambient noise levels controlled if performed on-site?

    Response: Testing was conducted in a sound-proof booth in our health facility; this has been captured in the methods section as well.

15. Line 156: List out the variables you are referring to.

    Response: We have now listed all variables (age, marital status, education, income, years of exposure, daily hours, family history, tinnitus, PTA outcome, OAE outcome) in the methods section.

RESULTS

16. Adding subheadings would help with organizing the presentation of results, beginning with the demographics and characteristics of the cohort.

    Response: Subheadings have now been added: *Demographics*, *Tinnitus Prevalence by Age*, *Cross-Tabulation of Hearing Outcomes*, *Regression Analysis*.

17. Reporting of the demographic characteristics needs to be improved and more comprehensive, in line with standard epidemiological reporting in studies. There should be a dedicated table for this. For age, please report the Ns across each decade encompassing the cohort/

    Response: A dedicated demographics table (Table 1) has now been added, reporting Ns and percentages across age decades and other variables.

18. The significance of 'marital status' could be a function of age. What was the mean age of the married vs. unmarried participants?

    Response: We thank the reviewer for this insightful observation/comment. However, during re-analysis and after adjustment, the marital-status association was fully explained by age and exposure duration, as such, marital status was not considered a meaningful explanatory variable in this study anymore.

19. In Table 1, please add footnotes with the definitions of "audiometric impairment", "OAE failure", and "Family History of Deafness"

    Response: Footnotes now define "audiometric impairment," "OAE failure," and "family history of deafness.

20. Line 191: Protective is not the right word. OAE performance (pass) is an indicator of better OHC health and function, i.e., lower levels of damage to the sensory cells that could be responsible for generating tinnitus. In this case, worse OAE function is associated with the presence of tinnitus.

    Response: Revised wording: *OAE pass indicates better outer hair cell function and lower risk of tinnitus, whereas OAE failure was associated with tinnitus presence.*

21. Table 2 is not needed and these findings can simply be reported in the text. If kept, "Other Covariates" need a footnote listing them out.

Response: Table 2 has been streamlined; age prevalence is now reported and supplemented with a LOESS curve figure 1.

22. Line 198-199: This statement does not seem to be true because there is a large increase (doubling) of the prevalence of tinnitus in participants age 25 y and younger versus those older than 25 y. This further suggests that the impact of age is not being sufficiently assessed.
    Response: We have added a table of tinnitus prevalence by age decade and a LOESS curve (Figure 1). Logistic regression of age (per 10-year increase) is also now reported figure 2.
23. In Table 4, the proportions of each group reporting tinnitus should be added.
    Response: Proportions have been added to Table 4 (now table 3), clarifying group differences.

DISCUSSION

24. Unless there is specific rationale for being labelled a pilot study, I suggest using 'cohort study.' Otherwise, please add the rationale somewhere in the Discussion.
    Response: Revised to *pilot cohort study*, with rationale explained (exploratory design, modest sample size).
25. Line 228-230: Prior studies reporting this finding should be mentioned/discussed.
    Response: We have added citations to the suggested studies and subtle discussed their relevance.
26. Lines 232-234: Regarding the statement about the integration of OAE screening in low-resources settings, isn't this already the case? It is routinely used for newborn screening with portable devices worldwide.
    Response: We can confirm that while OAE is NOT routinely used in newborn screening in our clime, its integration into occupational health screening in informal settings equally remains quite limited.
27. Line 243: The statement about the insignificance of age is not clear from the reported data. There should be a table tabulating tinnitus prevalence by age groups (e.g., decades) and plot it (age on x, % with tinnitus on y). Then fit a locally smoothed curve (e.g., LOESS) of prevalence vs. age to see shape. What did a logistic regression of age say (Model: $\text{logit}[P(\text{tinnitus}=1)] = \beta 0 + \beta 1 \cdot \text{Age}$). Report odds ratio per 10-year increase (scale age/10 so β1 is per decade).
    Response: We have mentioned earlier that age prevalence table, LOESS curve, and logistic regression results have been used to clarify age effects (see response to reviewer 1; comment 7).
28. Lines 247-249: Regarding the significance of marriage, what is the mean age of the married vs. unmarried men? Do the married men work longer hours/have more daily exposure due to supporting a family?
    Response: see response to comment 18.
29. Lines 262: Child labour is indeed a concerning feature of this cohort, so extra care need to be taken in the reporting of the informed consent. Was any counselling or education provided to participants? Were they made aware of the study results? Provided with hearing protection?
    Response: We clarified that minors too provided assent with parental consent, were also counselled on hearing risks, and offered protective advice (also see response to reviewer 2; comment 7).

30. Line 276-278: Please provide the rational for why the Tinnitus Functional Index or Tinnitus Handicap Inventory, etc. wasn't used? Lack of a translated version?
    Response: We explained that validated translations were unavailable locally, and resource constraints limited use of standardized instruments. A structured questionnaire was therefore employed.
