# Supplementary material for: Tinnitus and occupational noise exposure among informal generator technicians in Nigeria: A pilot cohort study
Source: PLOS Glob Public Health. 2026 Feb 26;6(2):e0005032. doi: 10.1371/journal.pgph.0005032 (PMC12944749; doi:10.1371/journal.pgph.0005032)
Supplement: Attachment — Submitted filename: Tinnnitus_Occupational Noise_ReviewerResponse_27012026.pdf [file pgph.0005032.s008.pdf]

Manuscript Title: PGPH-D-25-02022R2
Tinnitus and Occupational Noise Exposure among Informal Generator Technicians in Nigeria: A Pilot Cohort Study [PLOS Global Public Health]

Dear Helen Howard,
We thank you and the reviewers for your thoughtful and constructive feedback on this manuscript. We have carefully considered all comments and revised the manuscript accordingly. We have provided below a point-by-point response to the major concerns raised:

Reviewer #3: Congratulations for an informative manuscript, with conclusions that are largely well founded.

I have **two issues** which need addressing:

1. line 119: Please provide some more detail about the sample size calculation - for example was it designed to assess the prevalence of tinnitus, or to assess a particular magnitude of association of a categorical or continuous variable with tinnitus? If so, that is the power, effect size and type I error rate considered?

The reference here is indicating that in designing a study to detect a problem, then 60 patients is appropriate. The study in this manuscript is not a study to detect a problem, rather a study examining factors associated with tinnitus - thus it is inappropriate to justify the sample size with that reference. A statistician may be able to assist the author with justification of their sample size.
Response:
We thank the reviewer for raising this important point. We have clarified in the Methods that the initial target sample size was based on an assumed tinnitus prevalence of 20% in noise-exposed populations and a desired precision of ±10% at the 95% confidence level. This calculation yielded a required sample size of approximately 62. We then added 10% to account for potential non-response, giving a target of 66. Ultimately, 73 participants were recruited, which slightly improved the precision of the prevalence estimates. We have removed the previous reference with the "pilot study sample size rules" and replaced it with this more appropriate justification (line 119-124).

2. Figure 1/ Table 2 and table 3: please add 95% confidence intervals for the rates - to the numerical estimates and the graph. This helps to show the uncertainty of the estimates.

Response:
We appreciate the reviewer's suggestion to include confidence intervals, which indeed provide important information about the uncertainty of our estimates. We have now added 95% confidence intervals to the prevalence rates in Table 2 and to the odds ratios in Table 3. In addition, Figure 1 has been revised to display the 95% confidence intervals in-situ as vertical error bars. These changes improve the transparency and interpretability of our findings.

3. Typos:
line 219 - extra %. The extra % has now been corrected (line 239)
